# Expression of Congenital Anomalies of the Kidney and Urinary Tract (CAKUT) Candidate Genes *EDA2R*, *PCDH9*, and *TRAF7* in Normal Human Kidney Development and CAKUT

**DOI:** 10.3390/genes15060702

**Published:** 2024-05-28

**Authors:** Jelena Kelam, Nela Kelam, Natalija Filipović, Luka Komić, Anita Racetin, Dora Komić, Sandra Kostić, Ivana Kuzmić Prusac, Katarina Vukojević

**Affiliations:** 1 Department of Family Medicine, Split-Dalmatia County Health Center, 21000 Split, Croatia; jelena.kelam@dz-sdz.hr (J.K.); luka.komic@dz-sdz.hr (L.K.); 2 Department of Anatomy, Histology and Embryology, University of Split School of Medicine, 21000 Split, Croatia; nela.kelam@mefst.hr (N.K.); natalija.filipovic@mefst.hr (N.F.); amuic@mefst.hr (A.R.); dora.komic@mefst.hr (D.K.); sandra.kostic@mefst.hr (S.K.); 3 Department of Pathology, University Hospital Center Split, 21000 Split, Croatia; ivanakp@mefst.hr; 4 Department of Anatomy, School of Medicine, University of Mostar, 88000 Mostar, Bosnia and Herzegovina; 5 Center for Translational Research in Biomedicine, School of Medicine, University of Split, 21000 Split, Croatia

**Keywords:** CAKUT, *EDA2R*, *TRAF7*, *PCDH9*, congenital anomalies of the kidney and urinary tract

## Abstract

Approximately half of the cases of chronic kidney disease (CKD) in childhood are caused by congenital anomalies of the kidney and urinary tract (CAKUT). Specific genes were identified as having significant importance in regard to the underlying genetic factors responsible for the CAKUT phenotype, and in our research, we focused on analyzing and comparing the expression levels of ectodysplasin A2 receptor (EDA2R), protocadherin9 (PCDH9), and TNF receptor-associated factor 7 (TRAF7) proteins in the cortex and medulla of healthy control kidneys during developmental phases 2, 3, and 4. We also performed an analysis of the area percentages of the mentioned proteins in the cortical and medullary sections of healthy embryonic and fetal kidneys compared to those affected by CAKUT, including duplex kidneys (DK), horseshoe kidneys (HK), hypoplastic kidneys (HYP), and dysplastic kidneys (DYS). We found that the CAKUT candidate gene proteins EDA2R, PCDH9, and TRAF7 are all expressed during normal human kidney development stages. In DYS, the expression of EDA2R was higher than in normal kidneys, likely due to EDA2R’s role in apoptosis, which was upregulated in specific cases and could possibly contribute to the formation of DYS. The expression of PCDH9 was lower in HK, which can be attributed to the possible role of PCDH9 in cell migration suppression. Decreased PCDH9 expression is linked to increased cell migration, potentially contributing to the development of HK. The level of TRAF7 expression was reduced in all examined kidney disorders compared to normal kidneys, suggesting that this reduction might be attributed to the crucial role of TRAF7 in the formation of endothelium and ciliogenesis, both of which are essential for normal kidney development. Further research is required to ascertain the function of these proteins in both the typical development of the kidney and in CAKUT.

## 1. Introduction

The development of the urinary system is an intricate process that relies on precise spatiotemporally regulated events, and necessitates the integration of many progenitor cell populations from different embryonic sources [1,2]. This process requires delicate interactions between epithelial and mesenchymal cells, which ultimately result in the controlled development of specialized stromal, epithelial, and vascular cell types. This unique characteristic contributes to the complex architecture and functionality of the kidney [3]. 

The formation of nephrons, nephrogenesis, occurs prenatally, resulting in an estimated count of around one million nephrons per kidney at the time of birth, and may be divided into distinct morphological stages: renal vesicle (stage I), comma-shaped body, S-shaped body (stage II), capillary loop nephron (stage III), and maturing nephron (stage IV) [4,5]. Development of the metanephros can be divided into four phases, based on the branching pattern of the ureteric bud and its ability to induce nephron formation. In phase I (Ph1), which occurs from the 5th to approximately the 14th week of development, each tip (ampulla) of the branching ureteric bud induces the formation of one nephron. Phase II (Ph2) begins around the 15th week and concludes between the 20th and 22nd week of development. During this phase, the ureteric tree stops branching, and each tip induces the formation of several nephrons that attach to it in the form of an arcade. Then, the kidney enters the third phase (Ph3), which lasts until the 36th week of development. In this phase, each tip induces several nephrons, which all attach separately to the entire length of the collecting duct, and the majority of nephrons are formed during this phase. Lastly, during phase IV (Ph4), which begins at the 36th week of development and continues into adulthood, the tips disappear, and no new nephrons are formed while the existing nephrons mature [5,6]. Several external and internal factors can influence the disruption of nephrogenesis, leading to congenital anomalies in the kidney and urinary system [7,8]. 

Congenital anomalies of the kidney and urinary tract (CAKUT) are defined by the existence of structural and functional abnormalities that impact the kidney, collecting system, bladder, and urethra [9]. It is believed that they play a role in approximately 30% to 60% of cases of chronic kidney disease (CKD) occurring in childhood. According to the European Registry for Children on Renal Replacement Therapy (ESPN/ERA), in 2019, CAKUT was identified as the predominant etiology of renal failure in the pediatric population. This particular cause accounted for 40.7% of newly diagnosed cases of chronic kidney disease among children below the age of 15 who received kidney replacement therapy (KRT) [10]. It has been estimated that up to 20% of CAKUT cases can be attributed to an inherited etiology. Over 40 genomic disorders and 50 genes have been implicated as contributing to the etiology of both syndromic and nonsyndromic CAKUT [11]. Approximately 10% of sporadic instances can be attributed to mutations in a single gene. Furthermore, recent investigations have revealed that 15% of cases are linked to copy number variants (CNVs). CNVs refer to alterations in the number of copies of a specific DNA segment in the germline, with sizes typically ranging from 1 kilobase (kb) to several megabases (Mb). These changes represent the most extensive form of sequence variation observed in the human genome [12]. A list of five potential genes was found to be of high priority in relation to the underlying genetic variables responsible for the CAKUT phenotype. These genes include *DLG1*, *EDA2R*, *KIF12*, *PCDH9*, and *TRAF7* [13].

The ectodysplasin A2 receptor (EDA2R) belongs to the tumor necrosis factor receptor (TNFR) superfamily and specifically interacts with EDA-A2, a recently discovered protein that has a crucial function in the process of ectodermal differentiation [14]. It is produced by an alternative splicing isoform of the *EDA* gene (ectodysplasin A). The gene is situated on the Xq12 region of the chromosome, which is why it is also referred to as an X-linked ectodysplasin-A2 receptor (XEDAR) [15]. The EDA2R receptor has been recognized as a target of TP53, TP58, and EDA-A2 signaling [16]. Elevated levels of *EDA* were found in various metabolic disorders, including non-alcoholic fatty liver disease (NAFLD), obesity, and insulin resistance [17].

Protocadherins (PCDHs) are the biggest subgroup in the cadherin family, consisting of calcium-dependent cell–cell adhesion molecules. *Protocadherin 9* (*PCDH9*) is located at position 13q21.32 in the human genome, and is responsible for producing a protein expressed in a wide range of tissues [18,19]. Few studies have identified reduced expression of *PCDH9* as a potential novel risk gene for major depressive syndrome [20]. Prior research has shown that the expression of PCDH9 was reduced in non-nodal mantle cell lymphoma and glioblastoma due to changes in the number of gene copies. Additionally, it has been observed that introducing *PCDH9* externally can hinder the migration of tumor cells [21,22].

The tumor necrosis factor (TNF) and TNFR superfamilies include a collection of ligands and receptors, which can be either released or attached to the cell membrane. These receptors regulate various biological functions, such as apoptosis, osteoclastogenesis, and immune system control [23]. TNF receptor-associated factor 7 (TRAF7) has been discovered to be linked with MEKK3, a crucial signaling protein in the TNF-induced NF-kB activation pathway, which is vital for cell proliferation and survival. It also adds ubiquitin molecules to NEMO and p65, which are important components of the NF-kB pathway, leading to their destruction [24,25]. Identifying somatic *TRAF7* driver mutations has revealed their possible pathogenic role in various tumors, including meningiomas, mesothelioma, adenomatoid tumors, and intraneural perineuriomas [26,27,28,29].

*DLG1* and *KIF12* expression has already been investigated by Veljačić Visković et al., and they showed notable differences in the spatiotemporal expression of DLG1 and KIF12 during both prenatal and postnatal kidney development [30]. Previous findings made by our research team have made a significant breakthrough by identifying the expression of novel susceptibility genes *EDA2R*, *PCDH9*, and *TRAF7* in cases of CAKUT [13]. In this article, we aim to investigate the expression of these proteins in the normal developmental process of the kidney, and compare them with their expression in CAKUT to provide evidence of their potential usefulness for the diagnosis and treatment of CAKUT.

## 2. Materials and Methods

### 2.1. Procurement and Processing of Tissues 

Thirty-eight paraffin blocks containing embryonic and fetal kidney tissue (Table 1) were collected from the University Hospital Center Split’s department of pathology. The blocks originated from spontaneous miscarriages and eugenic abortions characterized by severe abnormalities. The processing of the blocks was authorized by the ethics and drug committee of the University Hospital in Split (class: 003-08/23-03/0015, approval number: 2181-198-03-04-23-0073), following the guidelines outlined in the Helsinki Declaration [31]. Examples of specimens not subjected to maceration were included in the investigation. The gestational age was estimated using menstrual data and external measurement (crown–rump length) [32]. The classification of renal pathology was based on routine histopathology and gross morphology.

Renal tissue was fixed in buffered formalin (4% paraformaldehyde in 0.1 M phosphate buffer saline, PBS) subsequent to the post-mortem section and embedded in paraffin blocks, as described previously [33,34]. The tissue was serially sectioned, and every 10th section was stained with hematoxylin–eosin (H&E) to confirm proper tissue preservation. Light microscopy was used to examine normal fetal kidney development stages and pathological findings in the CAKUT-affected kidneys.

### 2.2. Immunofluorescence

The immunofluorescence staining was performed according to the previously described protocol [33,35]. Following the application and overnight incubation of primary antibodies in a humidity chamber, secondary antibodies were added and incubated for one hour before being flushed with PBS (Table 2). DAPI (4′,6-diamidino-2-phenylindole) was utilized subsequent to rinsing in PBS to visualize nuclei. After rinsing in PBS, mounting media (Immuno-Mount, Thermo Shandon, Pittsburgh, PA, USA) and a coverslip were used to cover the slides.

Omitting primary antibodies in the immunofluorescence protocol did not result in the detection of nonspecific secondary antibody binding or false-positive results.

### 2.3. Data Acquisition

The H&E slides were examined using a light microscope (CX43, Olympus, Tokyo, Japan). An epifluorescence microscope (BX51, Olympus, Tokyo, Japan) with a Nikon DS-Ri2 camera (Nikon Corporation, Tokyo, Japan) and NIS-Elements F software were used to capture microphotographs of the embryonic and fetal kidney, namely the nephrogenic zone and juxtamedullary area of the cortex and medulla. EDA2R, PCDH9, and TRAF7 were examined in 10 distinct representative regions in the healthy control samples’ cortex and medulla (CTRL). As a result of the unique structure of the kidneys affected by CAKUT, it was challenging to differentiate between the cortex and medulla in some phenotypes, namely dysplastic kidneys (DYS). To address this issue, we captured a minimum of 20 visual fields in the group of kidneys afflicted by CAKUT. All photographs were captured using ×40 magnification and a consistent exposure period. Positive staining for EDA2R, PCDH9, and TRAF7 was observed as diffuse or punctate green staining.

### 2.4. Image Analysis of Area Percentage

The captured photographs were analyzed using ImageJ software version 1.54g (National Institutes of Health, Bethesda, MD, USA) to quantify the immunofluorescence signal of the observed proteins [33,36]. Each image was processed in the following way: Fluorescence leakage was minimized by removing the red countersignal from the green fluorescence. Next, a median filter with a radius of 7.0 pixels was applied. The non-filtered photos were subtracted from the filtered images to identify the positive signal, and the resulting pictures were converted into 8-bit images. Then, each image was sequentially modified using the triangle thresholding algorithm. The percentage of fluorescence area was determined using the “analyze particles” feature. In some regions of the images, the absence of tissue led to a lower calculated area percentage than the actual value. To correct this, the total number of pixels in the photos and the number of pixels representing empty space were computed using the Magic Wand tool in Adobe Photoshop version 21.0.2 (Adobe, San Jose, CA, USA). The corrected area percentage was determined by dividing the uncorrected area percentage multiplied by the total number of pixels less the number of pixels representing empty space, as previously described [37]. Then, this corrected area percentage was utilized for the statistical analysis. Accounting for differences in observations across observers, three skilled histologists independently examined the collected microphotographs and determined the background thresholds using negative control images. The interrater agreement was confirmed using interclass correlation analysis, which resulted in a coefficient greater than 0.8, suggesting a high level of agreement [38].

### 2.5. Statistical Analysis of Area Percentage

The statistical analysis was conducted using GraphPad Prism version 9.0.0 software (GraphPad Software in San Diego, CA, USA). The results are presented as the calculated percentages’ mean and standard deviation. The data’s distribution normalcy was assessed using the Shapiro–Wilk test. Each dataset pertaining to area percentage analysis was characterized by a probability level (*p*) of less than 0.05, which was considered statistically significant. 

We analyzed and compared EDA2R, PCDH9, and TRAF7 expression levels in the cortex and medulla of healthy control kidneys during developmental phases 2, 3, and 4 using an ordinary one-way analysis of variance (ANOVA) test followed by Tukey’s multiple comparison test. Developmental phase 1 was omitted from the analysis due to a limited number of photomicrographs caused by the extremely small tissue area, and the inability to adequately differentiate between the cortex and medulla of the embryonic and fetal kidney of the phase. 

Linear and nonlinear regression modeling was used to analyze observed proteins’ expression dynamics and trends during the four developmental phases of healthy control kidneys. To accomplish this, we calculated the average area percentage values of the kidney cortex and core of the samples representing different phases of development. A coefficient is defined as a point estimate ± standard error in trend description models. The coefficient of determination (R^2^) served as a metric to assess the goodness of fit. The slope of a linear regression line (β) was used to describe a linear trend. 

We conducted an analysis of the area percentages of cortical and medullary surface areas in embryonic and fetal kidneys, comparing healthy kidneys to those afflicted by CAKUT (duplex kidneys (DK), horseshoe kidneys (HK), hypoplastic (HYP) and dysplastic kidneys (DYS)) using an ordinary one-way ANOVA with Tukey’s post-hoc analysis.

The graphs were generated using GraphPad Prism 9.0.0. The plates were constructed using Adobe Photoshop 21.0.2 version. The microphotographs underwent background reduction and contrast enhancement to optimize their display.

## 3. Results

### 3.1. Hematoxylin and Eosin (H&E) of Normal Human Fetal Kidneys and Kidneys with Congenital Anomalies of the Kidney and Urinary Tract (CAKUT)

**Figure 1:**
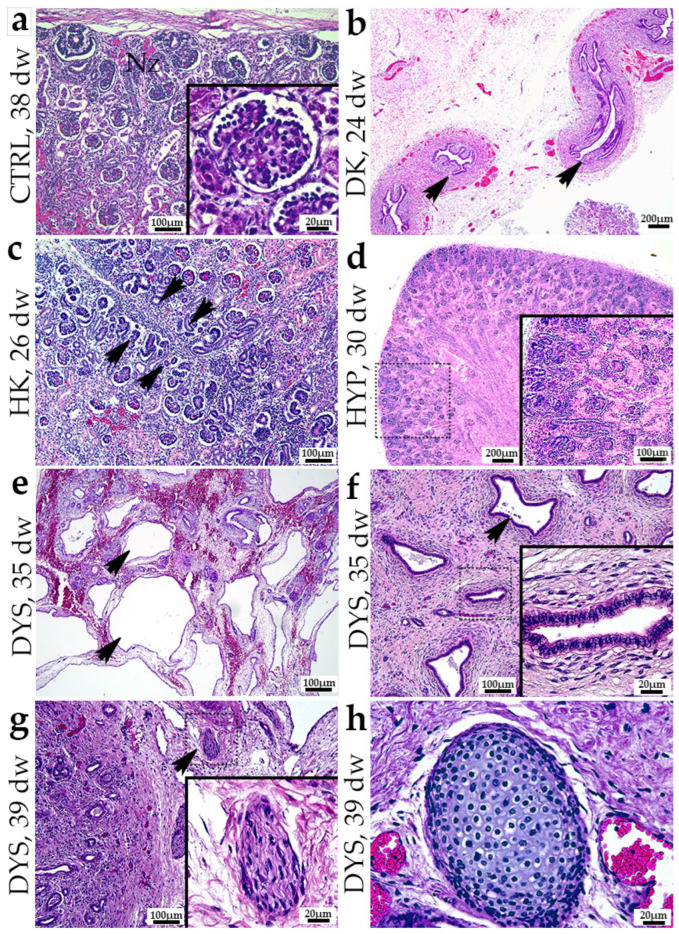
Hematoxylin and eosin images of normal fetal kidney at 38th dw (**a**) and kidneys affected with congenital anomalies: duplex kidney (DK) at 24th developmental week (dw) (**b**), horseshoe kidneys (HK) at 26 dw (**c**), hypoplastic kidneys (HYP) at 30th dw (**d**), and dysplastic kidney (DYS) at 35 dw (**e**,**f**) and 39 dw (**g**,**h**) (H&E). The most characteristic elements noticed for each observed phenotype are shown in inserts. A scale bar is indicated on each image.

The normal fetal kidney has a well-developed nephrogenic zone and podocytes displaying a cuboidal shape (Figure 1a). The duplex kidney displayed two ureters (Figure 1b). Although the microscopic imaging does not display the specific region, the renal parenchyma in the duplex kidney is normal. The horseshoe kidney displayed two nephrogenic zones consisting of a primitive basophilic band where the formation of glomeruli and tubules occurs (Figure 1c). The hypoplastic kidney exhibits a normal kidney structure with a narrower nephrogenic zone compared to a normal kidney. Additionally, it is characterized by a reduced number of nephrons (Figure 1d). Dysplastic kidneys exhibit aberrant differentiation of nephrons and renal tubules, consisting of atypically formed metanephric components with an irregular structural arrangement, as opposed to the typical nephrogenesis process. Kidneys diagnosed with dysplasia exhibit cysts originating from primitive ducts (Figure 1e). The ducts exhibited a variety of shapes, ranging from round to oval. Some of them had a curved form. The lining of the ducts was mostly composed of tall columnar cells surrounded by a fibromuscular collar. Additionally, there was evidence of lobar disorganization (Figure 1f). The rudimentary ducts emerged individually or in tiny clusters within a defined lobule consisting of elongated, connective tissue cells. Dysplastic kidneys included unmyelinated nerve fibers within the mesenchyme (Figure 1g). Furthermore, distinct areas of properly developed cartilage were seen (Figure 1h).

### 3.2. EDA2R Expression

**Figure 2:**
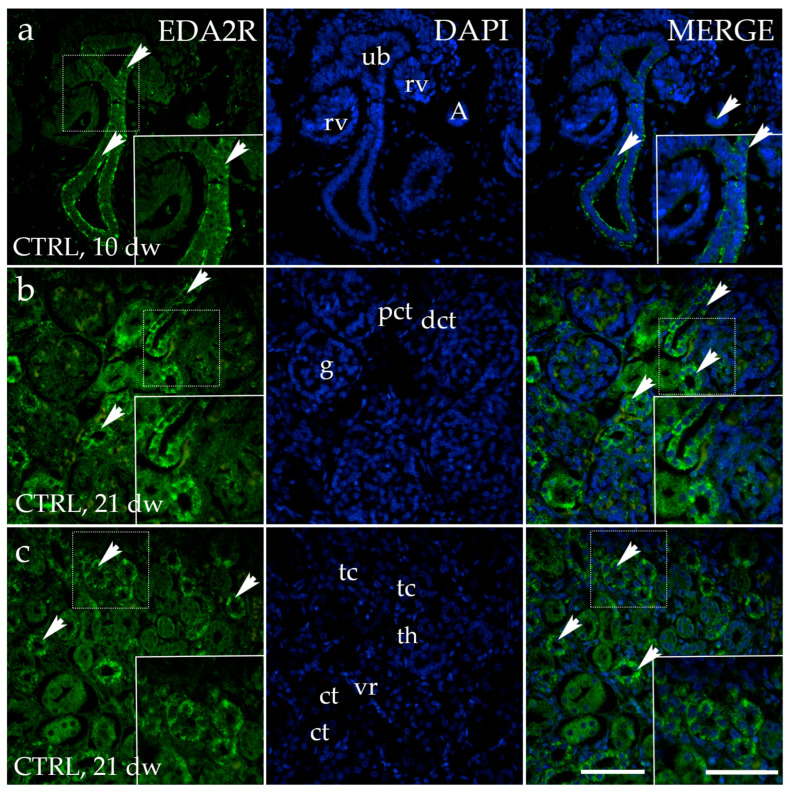
Immunofluorescence staining of the renal cortex (**a**,**b**) and medulla (**c**) of normal human fetal kidney at the 10th and 21st developmental week (dw) (CTRL) with the antibody for ectodysplasin A2 receptor (EDA2R). The arrows indicate the pattern of expression of EDA2R in the ureteric bud (ub), ampulla (A), renal vesicle (rv), glomeruli (g), proximal convoluted tubules (pct), and distal convoluted tubules (dct) of the renal cortex, and vasa recta (vr), collecting tubules (ct), and thick (tc) and thin (th) segments of the loop of Henle of the renal medulla indicated on the nuclear 4′,6-diamidino-2-phenylindole staining image (DAPI). Immunoexpression of merged EDA2R and DAPI in normal human fetal kidney (MERGE). The inserts that match the dashed boxes indicate the major region of protein expression. The scale bar is 50 μm for all images.

EDA2R exhibited a strong punctative signal in the apical and basal membranes of the ureteric bud cells of the developing kidney at developmental phase 1 (Ph1) (Figure 2a). S-shaped, comma-shaped bodies, and renal vesicles displayed no staining (Figure 2a). In later developmental phases, a diffuse cytoplasmic signal was detected in the juxtaglomerular region of a healthy kidney. The apparent location of EDA2R is strongest in the distal and slightly lower in the proximal convoluted tubule and glomerulus (Figure 2b). EDA2R is prominently stained within the thick segment of the loop of Henle in the renal medulla. We did not notice any staining in the collecting tubules (Figure 2c). 

**Figure 3:**
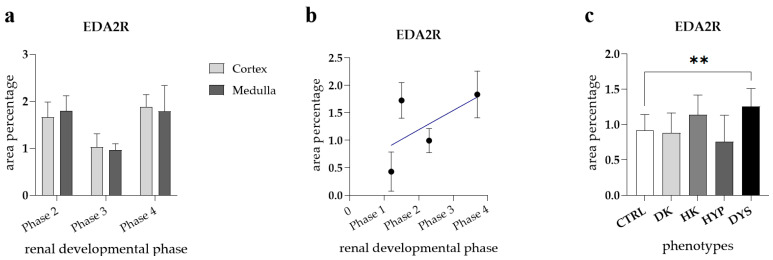
The ectodysplasin A2 receptor (EDA2R) area percentages in the cortex and medulla of normal fetal kidney tissues during developmental phases 2, 3, and 4 (**a**). Data are presented as the mean ± SD (vertical line) and analyzed via an ordinary one-way ANOVA test followed by Tukey’s multiple comparison test. At each developmental phase, ten representative pictures were assessed per observed region. The expression dynamics of EDA2R (**b**) showed by linear and nonlinear regression modeling of area percentages through developmental phases in the cortex and medulla of CTRL fetal kidney tissues. Data are presented as the mean ± SD. The EDA2R area percentages in the duplex kidneys (DK), horseshoe kidneys (HK), hypoplastic kidneys (HYP), and dysplastic kidneys (DYS) fetal tissue (**c**). Data are presented as the mean ± SD (vertical line) and analyzed via an ordinary one-way ANOVA test followed by Tukey’s multiple comparison test. Significant differences are indicated by ** *p* < 0.01. At each time point, twenty representative pictures were assessed.

No significant difference was observed when comparing the area percentage of EDA2R-positive cells between the cortex and medulla of healthy fetal kidneys across examined developmental phases (Figure 3a). When formally tested for a linear trend among observed developmental phases, no significant difference was found (R^2^ = 26.62%, β = 0.03501 ± 0.01319, Figure 3b).

**Figure 4:**
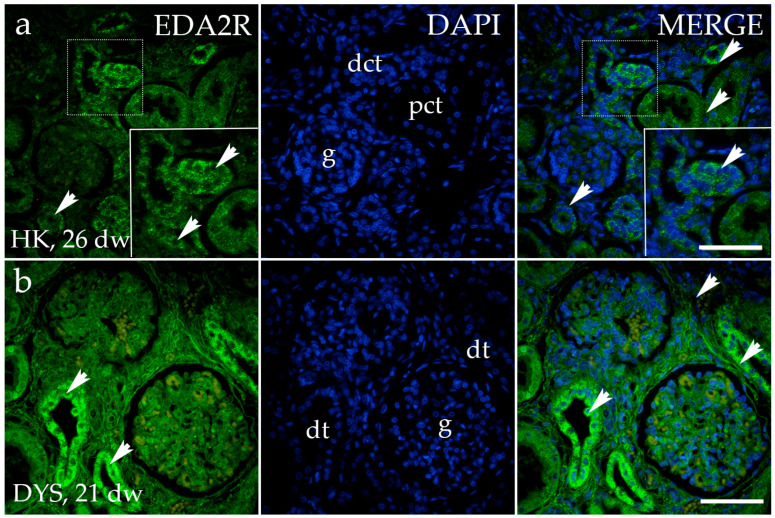
Immunofluorescence staining of human fetal kidneys with the antibodies for ectodysplasin A2 receptor (EDA2R) (**a**,**b**). The arrows indicate the pattern of expression of EDA2R in glomeruli (g), proximal convoluted tubules (pct), distal convoluted tubules (dct), and dysplastic tubules (dt), indicated on the nuclear 4′,6-diamidino-2-phenylindole staining image (DAPI). The staining patterns of EDA2R in HK, DK, and HYP mostly correspond in terms of their localization and intensity; thus, a representative image was selected from the horseshoe kidney sample at the 26th dw. Immunoexpression of EDA2R and DAPI staining and merged EDA2R and DAPI in the horseshoe kidneys (HK) at 26th dw (**a**) and dysplastic kidney (DYS) at 21st dw (**b**). The inserts that match the dashed boxes indicate the major region of protein expression. The scale bar is 50 μm for all images.

The staining patterns of the EDA2R in HK, DK, and HYP mostly correspond to their localization and intensity (Figure 4a). EDA2R is strongly expressed in dysplastic tubules in the dysplastic kidney, and is weaker in the glomerulus and surrounding connective tissue (Figure 4b). Comparison of the area percentage of EDA2R-positive cells between control and kidneys affected with CAKUT revealed a notable decrease in the percentage of EDA2R-positive cells in the control kidneys compared to DYS (*p* < 0.01, Figure 3c).

### 3.3. PCDH9 Expression

**Figure 5:**
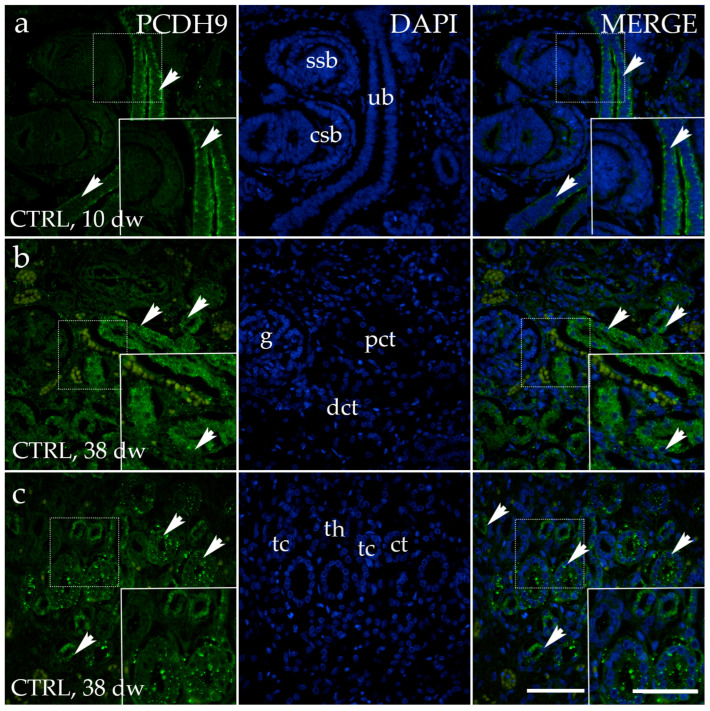
Immunofluorescence staining of the renal cortex (**a**,**b**) and medulla (**c**) of normal human fetal kidney at the 10th and 38th developmental weeks (dws) (CTRL) with the antibody for protocadherin 9 (PCDH9). The arrows indicate the pattern of expression of PCDH9 in the ureteric bud (ub), comma-shaped body (csb), s-shaped body (ssb), glomeruli (g), proximal convoluted tubules (pct) and distal convoluted tubules (dct) in the renal cortex, collecting tubules (ct), and thick (tc) and thin (th) segments of the loop of Henle of the renal medulla indicated on the nuclear 4′,6-diamidino-2-phenylindole staining image (DAPI). Immunoexpression of merged PCDH9 and DAPI in normal human fetal kidney (MERGE). The inserts that match the dashed boxes indicate the major region of protein expression. The scale bar is 50 μm for all images.

The spatiotemporal staining pattern of PCDH9 was similar to EDA2R and can be detected as a punctuative green signal, visible in the apical and basal membranes of the ureteric bud cells of the healthy kidney at Ph1. Similarly, the comma- and s-shaped bodies exhibited no staining (Figure 5a). PCDH9 exhibited moderate punctuative cytoplasmic staining in the tubules of the cortex, and a pronounced granular staining pattern in the collecting tubules of the renal medulla (Figure 5c). Thick and thin segments of the loop of Henle demonstrated a punctative cytoplasmic staining pattern (Figure 5c). 

**Figure 6:**
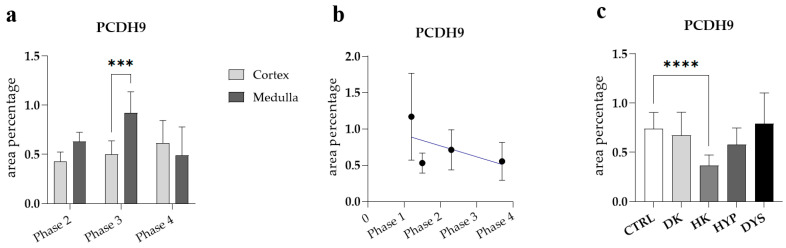
The protocadherin 9 (PCDH9) area percentages in the cortex and medulla of normal fetal kidney tissues during developmental phases 2, 3, and 4 (**a**). Data are presented as the mean ± SD (vertical line) and analyzed via an ordinary one-way ANOVA test followed by Tukey’s multiple comparison test. Significant differences are indicated by ***, *p* < 0.001. At each developmental phase, ten representative pictures were assessed per observed region. The expression dynamics of PCDH9 (**b**) showed by linear and nonlinear regression modelling of area percentages through developmental phases in the cortex and medulla of CTRL fetal kidney tissues. Data are presented as the mean ± SD. The PCDH9 area percentages in the duplex kidneys (DK), horseshoe kidneys (HK), hypoplastic kidneys (HYP), and dysplastic kidneys (DYS) fetal tissue (**c**). Data are presented as the mean ± SD (vertical line) and analyzed by an ordinary one-way ANOVA test followed by Tukey’s multiple comparison test. Significant differences are indicated by **** *p* < 0.0001. At each time point, twenty representative pictures were assessed.

A comparison of the percentage of PCDH9-positive cells in the medulla and cortex of healthy fetal kidneys during developmental phase 3 revealed a substantially higher rate in the medulla than in the cortex (Figure 6a). No statistically significant difference was identified when a formal test for a linear trend among the observed developmental phases was conducted (R^2^ = 26.62%, β = −0.01506 ± 0.00957, Figure 6b).

**Figure 7:**
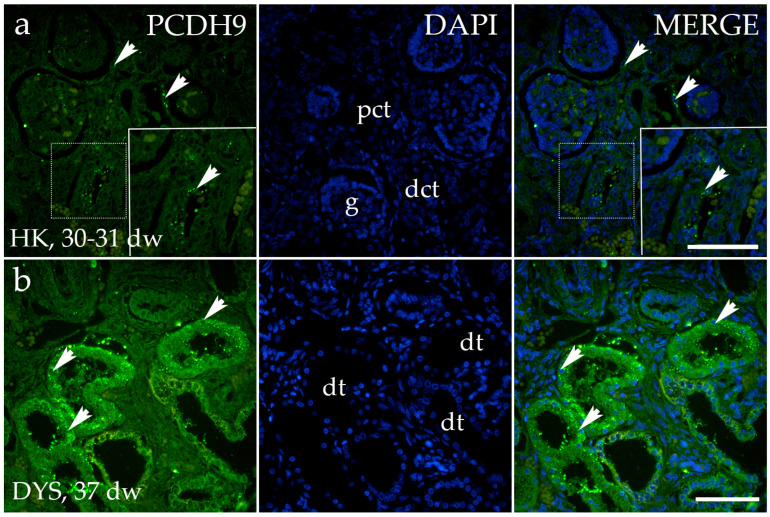
Immunofluorescence staining of human fetal kidneys with the antibodies for protocadherin 9 (PCDH9) (**a**,**b**). The arrows indicate the pattern of expression of PCDH9 in glomeruli (g), proximal convoluted tubules (pct), distal convoluted tubules (dct), and dysplastic tubules (dt), indicated on the nuclear 4′,6-diamidino-2-phenylindole staining image (DAPI). The PCDH9 staining patterns in CTRL, DK, and HYP correspond largely in their localization and intensity; therefore, a representative image was chosen from the horseshoe kidney sample of the 30–31st dw. Immunoexpression of PCDH9 and DAPI staining and merged PCDH9 and DAPI in the horseshoe kidneys (HK) at 30–31st dw, (**a**) and dysplastic kidney (DYS) at 37th dw (**b**) (Figure 5a). The inserts that match the dashed boxes indicate the major region of protein expression. The scale bar is 50 μm for all images.

The PCDH9 staining patterns in CTRL, DK, and HYP correspond largely to their localization and intensity (Figure 7a). PCDH9 is highly expressed in the tubules of a dysplastic kidney with an observed granular staining pattern (Figure 7b). There was an absence of staining in the connective tissue and the dysplastic kidney’s glomeruli. An analysis of the percentage of PCDH9-positive cells in the control kidneys and kidneys affected with CAKUT demonstrated a significant reduction in the proportion of PCDH9-positive cells in the control kidneys when compared to the horseshoe kidney (*p* < 0.0001, Figure 6c).

### 3.4. TRAF7 Expression

**Figure 8:**
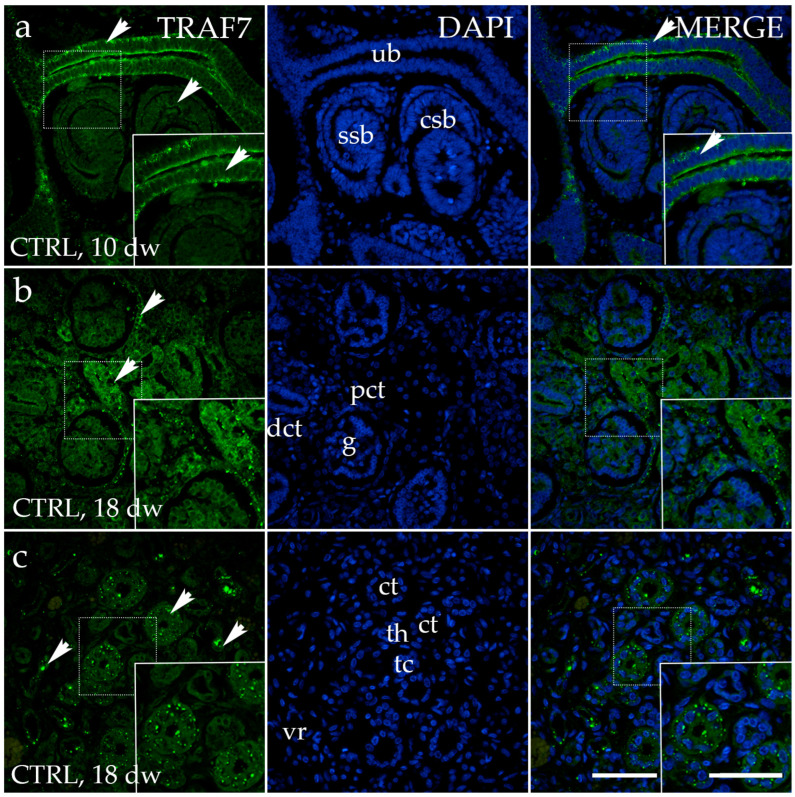
Immunofluorescence staining of the renal cortex (**a**,**b**) and medulla (**c**) of normal human fetal kidney at the 10th and 18th developmental weeks (dws) (CTRL) with the antibody for TNF receptor-associated factor 7 (TRAF7). The arrows indicate the pattern of expression of TRAF7 in the ureteric bud (ub), comma-shaped body (csb), s-shaped body (ssb), glomeruli (g), proximal convoluted tubules (pct) and distal convoluted tubules (dct) in the renal cortex, and vasa recta (vr), collecting tubules (ct), and thick (tc) and thin (th) segments of the loop of Henle of the renal medulla indicated on the nuclear 4′,6-diamidino-2-phenylindole staining image (DAPI). Immunoexpression of merged TRAF7 and DAPI in normal human fetal kidney (MERGE). The inserts that match the dashed boxes indicate the major region of protein expression. The scale bar is 50 μm for all images.

The immunohistochemical staining pattern of TRAF7 was detected as a distinct diffuse cytoplasmic green signal, more prominently expressed on the apical side of ureteric bud cells of normal kidneys at developmental phase 1 (Figure 8a). No staining was observed in the comma- and s-shaped bodies. Uniform diffuse cytoplasmic staining was detected in all visible components of the nephrogenic zone of the kidney cortex, including the glomerulus and distal convoluted tubule. Later developmental phases exhibit positive signals in the parietal layer of the renal corpuscle and the proximal convoluted tubule of the juxtamedullary region of the renal cortex (Figure 8b). TRAF7 has a distinct and intense staining pattern in the collecting tubules, and is evenly distributed in the thick segment of the loop of Henle within the kidney’s medulla (Figure 8c). 

**Figure 9:**
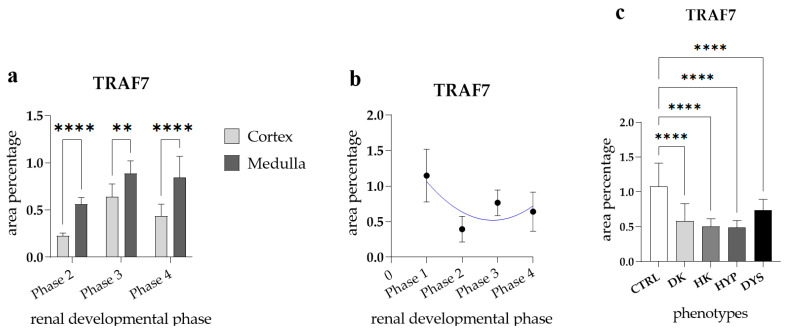
The TNF receptor-associated factor 7 (TRAF7) area percentages in the cortex and medulla of normal fetal kidney tissues during developmental phases 2, 3, and 4 (**a**). Data are presented as the mean ± SD (vertical line) and analyzed via an ordinary one-way ANOVA test followed by Tukey’s multiple comparison test. Significant differences are indicated by ** *p* < 0.01, **** *p* < 0.0001. At each developmental phase, ten representative pictures were assessed per observed region. The expression dynamics of TRAF7 (**b**) showed by linear and nonlinear regression modeling of area percentages through developmental phases in the cortex and medulla of CTRL fetal kidney tissues. Data are presented as the mean ± SD. The TRAF7 area percentages in the duplex kidneys (DK), horseshoe kidneys (HK), hypoplastic kidneys (HYP), and dysplastic kidneys (DYS) fetal tissue (**c**). Data are presented as the mean ± SD (vertical line) and analyzed via an ordinary one-way ANOVA test followed by Tukey’s multiple comparison test. Significant differences are indicated by **** *p* < 0.00001. At each time point, twenty representative pictures were assessed.

The medulla of healthy fetal kidneys displayed a significantly higher area percentage of TRAF7-positive cells compared to the cortex in all observed developmental phases (*p* < 0.0001, Figure 9a). The expression of TRAF7 in the healthy kidneys throughout the observed developmental phases followed a quadratic trend, with the peak at Ph1 (R^2^ = 29%, Figure 9b).

**Figure 10:**
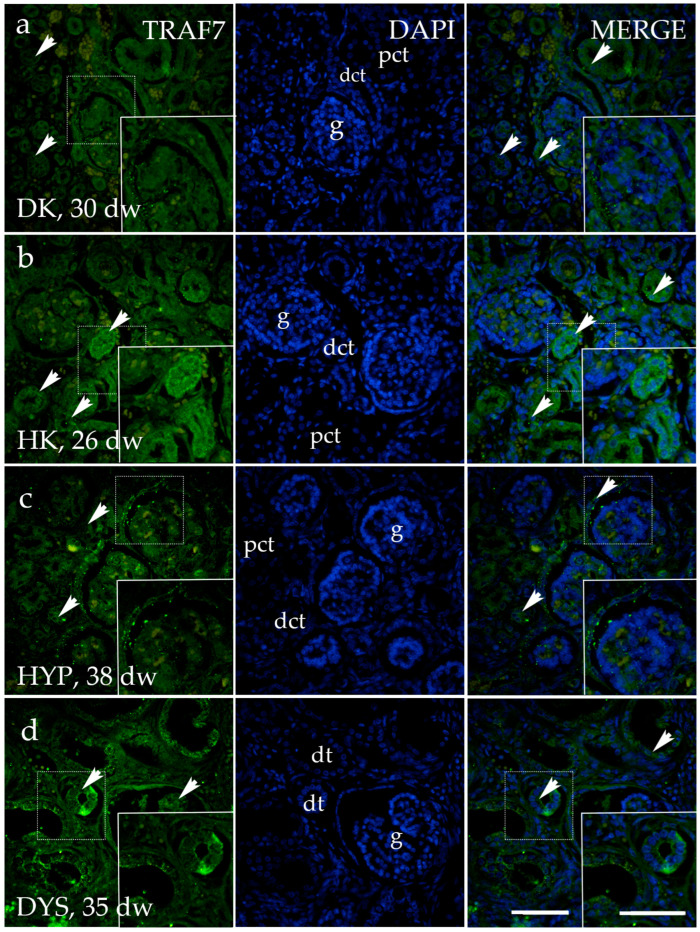
Immunofluorescence staining of human fetal kidneys with the antibodies for TNF receptor-associated factor 7 (TRAF7) (**a**–**d**). The arrows indicate the pattern of expression of TRAF7 in glomeruli (g), proximal convoluted tubules (pct), distal convoluted tubules (dct), and dysplastic tubules (dt), indicated on the nuclear 4′,6-diamidino-2-phenylindole staining image (DAPI). Immunoexpression of TRAF7 and DAPI staining and merged TRAF7 and DAPI in the duplex kidney (DK) at 30th dw (**a**), horseshoe kidneys (HK) at 26th dw, (**b**), hypoplastic kidney (HYP) at 38th dw (**c**), and dysplastic kidney (DYS) at 35th dw (**d**). The inserts that match the dashed boxes indicate the major region of protein expression. The scale bar is 50 μm for all images.

The staining patterns of the TRAF7 in HK, DK, and HYP mostly correspond to their localization and intensity (Figure 10a–c). TRAF7 is strongly expressed in dysplastic tubules in the dysplastic kidney, and is weaker in the glomerulus and surrounding connective tissue (Figure 10d). 

Comparison of the area percentage of TRAF7-positive cells between control and kidneys affected with CAKUT revealed a notable increase in the percentage of TRAF7-positive cells in the control kidneys compared to all other observed phenotypes (DK, HK, HYP, and DYS) (*p* < 0.0001, Figure 9e).

## 4. Discussion

This study focuses on investigating the spatial and temporal expressions of three candidate gene proteins—EDA2R, PCDH9, and TRAF7—which were identified by the in silico KIMONO study as potential genes that could be altered in CAKUT-related disorders [13]. To the best of our knowledge, the expression of these proteins in both normal human or animal kidney development and in patients diagnosed with CAKUT has not been described previously. Our hypothesis is that these proteins play a crucial role in proper kidney development, as evidenced by their distinct expression patterns during various phases of development. Additionally, any alterations in the expressions of these proteins could potentially lead to CAKUT-associated disorders. The results of our analysis revealed statistically significant disparities among developmental pathways, which could aid in identifying the critical period during which *EDA2R*, *TRAF7*, and *PCDH9* regulate appropriate renal maturation.

EDA2R showed a statistically higher area percentage in the renal dysplasia specimens compared to the control group. *EDA2R* can stimulate the JNK and NF-kB pathways, which results in inflammation and apoptosis [39,40]. It has previously been described that stimulation of *ED2AR* can lead to the apoptosis of various cell types, including cancer cells, salivary gland epithelial cells, osteosarcoma cell lines, and hair follicle cells [41,42]. Apoptosis has an important role in kidney development, and if the process of apoptosis is altered, developmental diseases could occur [43]. A study performed by Winyard et al. found a significantly increased incidence of apoptosis in cells of dysplastic human kidneys compared to age-matched normal controls [44]. Therefore, the increased expression of EDA2R in our dysplastic kidney samples could potentially be associated with the increased rates of apoptosis described in dysplastic kidneys. Further studies are necessary to better define the association between *EDA2R* and apoptosis in kidney development and disorders. Additionally, it has been demonstrated that overexpression of *EDA2R* can lead to the generation of reactive oxygen species and podocyte apoptosis in diabetic nephropathy [17,45].

Our results showed lower PCDH9 expression in the horseshoe kidney (HK) samples compared to the control group. This could be explained by the study conducted by Zhu et al., which described that *PCDH9* inhibits cell migration by activating GSK-3β, inhibiting tumor invasion in hepatocellular carcinoma [46]. Decreased *PCDH9* expression might potentially facilitate aberrant cell migration across the sagittal plane and the merging of renal cells at their distal end. This ectopic nephrogenic tissue is believed to be caused by the incomplete or aberrant movement of posterior nephrogenic cells over the primordial streak [47,48,49]. This abnormal cell migration could also be responsible for the varying compositions of the HK isthmus, which comprises predominantly renal parenchyma (80% to 85%) or fibrous tissue [46]. Zhu et al. also proposed that *PCDH9* inhibits epithelial–mesenchymal transition (EMT) in hepatocellular carcinoma by inhibiting Snail1 [46]. Interestingly, EMT also occurs normally during development without leading to tumor formation. In kidney development, the ureteric bud, an epithelial structure, is formed at an early stage of development through mesenchymal–epithelial transition of the intermediate mesoderm, while cells of the metanephric mesenchyme express Snail1 and remain mesenchymal. After induction by the ureteric bud, cells of the metanephric mesenchyme downregulate Snail1 expression, organize themselves around branching ureteric buds, coalesce into aggregates, and epithelialize to form renal vesicles [50,51,52]. The excessive growth of the metanephric blastema in HK may be associated with decreased *PCDH9* expression, and consequently decreased Snail1 downregulation. Additional studies are needed to elucidate the role of *PCDH9* in EMT during nephrogenesis and in renal pathology. 

Our study revealed that the level of TRAF7 immunoexpression is consistently higher in the medulla than in the cortex throughout all phases of kidney development. This distribution of TRAF7 was also detected in samples of healthy adult kidneys [53]. A recent study demonstrated that *Traf7* is linked to proper endothelium formation, and murine embryos lacking *Traf7* died in the uterus at E10 as a result of malfunctioning endothelial cells [54]. The kidneys, being highly vascularized organs, can be significantly impacted by endothelial dysfunction [55]. Previous research has demonstrated that endothelial cells exhibit synchronized growth with the ureteric bud in mice as early as embryonic day 10.5, equivalent to a human embryo’s fourth week of gestation. By embryonic day 13.5, these cells exhibit signs of differentiation, corresponding to the initial detection of arteries [56]. In the context of a human embryo, these events occur during Ph1 of kidney development. Ph3 of kidney development represents the period when most of the nephrons are induced and subsequently vascularized during the capillary loop stage, when endothelial cells undergo significant flattening and develop large fenestrations [5,57,58]. While the formation of the nephron and endothelium occurs during all stages of kidney development, the highest area percentage of TRAF7 immunoexpression, as indicated by our data, is observed during Ph1 and Ph3. Therefore, it can be inferred that a deficiency in TRAF7 is most likely to result in pathological conditions during these specific phases.

Our research demonstrated that the area percentage of TRAF7 in all pathological kidneys was significantly lower compared to the CTRL group. Mishra-Gorur et al. conducted a study to examine the impact of *Traf7* deletion in living organisms utilizing Xenopus and zebrafish models [59]. They discovered that loss of *Traf7* resulted in cardiac looping abnormalities, pericardial edema, and varied degrees of craniofacial deformities. Furthermore, they demonstrated that the depletion of *Traf7* resulted in impaired ciliogenesis with alterations in the morphology, spatial arrangement, and number of both primary and motile cilia [59]. Primary cilia play a vital role in the development of the kidney and the regulation of epithelial cell differentiation and growth. When these cilia are dysfunctional, it can result in various illnesses affecting several organs, including the kidneys. These disorders are known as ciliopathies [60]. The cilium is composed of many proteins transported throughout the cilia by the intraflagellar transport (IFT) particle system, which is necessary for the maintenance and expansion of cilia, as protein synthesis does not occur within the cilia [61]. Mishra-Gorur et al. discovered that *Traf7* interacts with the protein IFT57, which is involved in intraflagellar transport. When *Traf7* was suppressed in Xenopus tropicalis, it resulted in a disruption of intraflagellar transport [60]. The absence or impaired function of cilia is linked to certain kidney disorders and syndromes, including autosomal dominant polycystic kidney disease, autosomal recessive polycystic kidney disease, Bardet–Biedl syndrome, nephronophthisis, Senior–Loken syndrome, Joubert syndrome, and Meckel–Gruber syndrome [62]. Kakkar et al. demonstrated that almost 50% of the dysplastic kidneys observed were diagnosed as cases of multicystic dysplasia [63]. Our research findings showed a significant decrease in the area percentage of TRAF7 in all types of diseased kidneys, including those with dysplasia. This suggests that one possible explanation for the link between *TRAF7* deficiency and these conditions could be that *TRAF7* downregulation might possibly induce primary cilia dysfunction. However, additional studies are needed to confirm the indicated concept and explore the relationship between *TRAF7* and primary cilia in renal pathologies. 

Finally, *TRAF7* also triggers apoptosis through many routes, including enhancing the activation of MEKK3-induced AP1, NF-κB, and C/EBP-homologous protein, as well as inducing caspase-dependent apoptosis [24,64]. As previously stated, the dysregulation of apoptosis is a significant factor that impacts organogenesis [43].

The main limitation of our study is its observational nature. Due to the fact that our samples are archived human fetal material that was paraffin-embedded and formalin-fixed, we could not perform procedures for quantitative protein expression analysis such as flow cytometry or Western blotting. With this in mind, our results are valuable as they demonstrate the changes in EDA2R, PCDH9, and TRAF7 immunoexpression during normal human kidney development and in renal disorders. Additional observational studies on human material and experimental studies on animal models are needed to further our understanding of the role of the analyzed proteins in normal kidney development and CAKUT. 

## 5. Conclusions

The expression of CAKUT candidate gene proteins EDA2R, PCDH9, and TRAF7 is present throughout all phases of normal human kidney development. Compared to normal kidneys, the expression of EDA2R was increased in dysplastic kidneys, while the expression of PCDH9 was decreased in HKs. The expression of TRAF7 was decreased in all analyzed kidney disorders compared to normal kidneys. Additional studies are needed to determine the role of these proteins during normal kidney development and in CAKUT.

## Figures and Tables

**Table 1:** The samples of human embryonic and fetal kidneys (*n* = 38) analyzed in this study.

Groups	Developmental Phase	Renal and Associated Pathology	Gestational Phases	Number of Kidney Samples
Normal kidneys (CTRL)	Ph1	N/A	6	1
		N/A	10	1
		N/A	12	1
	Ph2	N/A	15	1
		N/A	16	1
		N/A	17	1
		N/A	18	2
		N/A	21	1
	Ph3	N/A	23	1
		N/A	24	1
		N/A	28	3
		N/A	29	1
	Ph4	N/A	32	1
		N/A	35	1
		N/A	37	1
		N/A	38	2
Horseshoe kidneys (HK)	*Ren concreatus arcuatus*, *cystae multiplices corticales*		22	1
	*Ren concreatus arcuatus*, *tetras Fallot*		26	1
	*Syndroma Edwards*, *Ren arcuatus*		30–31	1
	*Syndroma Edwards*, *Ren arcuatus*		34	1
Dysplastic kidneys (DYS)	*Megaureter lateris dextri*, *Dysplasia renis*		21	2
	*Renis dysplastica cysticus* *lateralis sinistri*, *agenesia renis dextri*			
	*Dysplasia multicystica renis dextri*		27	1
	*Cystes parvae focales*			
	*Renes dysplastici cystici*, *Syndroma Potter*		35	2
	*Dysplasia renis multicystica* *bilateralis*			
	*Agenesis renis dextri et dysplasia renis sinistri cum ureter duplex*		37	1
	*Dysplasia hypoplastica*, *renis bilateralis, syndroma Down*, *syndroma Potter*		38	1
	*Syndroma Potter*, *Dysplasia renis*		39	1
Hypoplastic kidneys (HYP)	*Hypoplasia renis lateris dextri*		30	1
	*Hypoplasia renis lateris sinistri*		37	1
	*Hypoplasia renis*		38	1
Duplex kidneys (DK)	*Ureter duplex lateris dextri*		24	1
	*Ureter duplex lateris sinistri*		30	1
	*Pyelon et ureter duplex bilateralis*		41	1

**Table 2:** Primary and secondary antibodies used for immunofluorescence.

Antibodies		Catalog Number	Host	Dilution	Source
Primary	Anti-EDA2R/XEDAR antibody	ab203667	Rabbit	1:120	Abcam (Cambridge, UK)
	PCDH9 Polyclonal antibody	25090-1-AP	Rabbit	1:200	Proteintech Group, Inc. (Rosemont, IL, USA)
	TRAF7 Polyclonal antibody	11780-1-AP	Rabbit	1:50	Proteintech Group, Inc. (Rosemont, IL, USA)
Secondary	Anti-Rabbit IgG, Alexa Fluor ^®^ 488	711-545-152	Donkey	1:300	Jackson Immuno Research Laboratories, Inc. (Baltimore, PA, USA)

## Data Availability

All data and materials are available upon request.
